# N-acetylcysteine suppresses colistimethate sodium-induced nephrotoxicity via activation of SOD2, eNOS, and MMP3 protein expressions

**DOI:** 10.1080/0886022X.2018.1489286

**Published:** 2018-07-23

**Authors:** Bahadir Ceylan, Mehmet Ozansoy, Ülkan Kılıç, Yasemin Yozgat, Çilem Ercan, Pelin Yıldız, Turan Aslan

**Affiliations:** a Department of Infectious Diseases and Clinical Microbiology, Faculty of Medicine, Istanbul Medipol University, Istanbul, Turkey;; b Department of Physiology, International School of Medicine, Istanbul Medipol University, Istanbul, Turkey;; c Department of Medical Biology, Faculty of Medicine, University of Health Sciences, Istanbul, Turkey;; d Department of Medical Biology, Faculty of Medicine, Istanbul Medipol University, Istanbul, Turkey;; e Department of Medical Biology, Faculty of Medicine, Bezmialem Vakıf University, Istanbul, Turkey;; f Department of Pathology, Faculty of Medicine, Bezmialem Vakıf University, Istanbul, Turkey,; g Department of Infectious Diseases and Clinical Microbiology, Faculty of Medicine, Bezmialem Vakıf University, Istanbul, Turkey

**Keywords:** Antioxidant, colistin, nephrotoxicity, rat model, reactive oxygen species

## Abstract

**Objective:** To investigate the molecular mechanisms of colistimethate sodium-induced nephrotoxicity and the protective effect of N-acetylcysteine (NAC) against nephrotoxicity.

**Methods:** Twenty-eight Wistar rats were divided into four groups comprised of control, colistin, NAC, and colistin–NAC co-treatment, respectively. Serum creatinine and urine N-acetyl-β-d-glucosaminidase (NAG) levels were measured at different time intervals. Histological changes, apoptosis, total oxidant and antioxidant status, and the expression levels of endothelial nitric oxide synthase (eNOS), superoxide dismutase 2 (SOD2), and matrix metalloproteinase 3 (MMP3) were evaluated in renal tissue.

**Results:** In the colistin group, post-treatment creatinine levels were higher than pretreatment levels (*p* = .001). There was a significant increase in urine NAG level following colistin treatment on day 10, compared to the baseline value and the first day of treatment (*p* = .001 and .0001, respectively). Urine NAG levels were higher in the colistin group on the 10th day of treatment than in the other groups (*p* < .01). Colistin treatment increased the apoptosis index and renal histological damage score (RHDS) significantly and these changes were reversed in NAC co-treatment (RHSD and apoptosis index were 45 and 0 for sterile saline group, 29 and 2 for NAC group, 122 and 7 for colistin group, and 66 and 2 for colistin + NAC group). We observed no difference between groups regarding total antioxidant and total oxidant status in the kidneys. The expression levels of eNOS, SOD2, and MMP3 decreased significantly in the kidneys of colistin-treated rats; these changes were reversed in the kidneys of NAC co-treated rats.

**Conclusions:** N-acetylcysteine prevented colistin-induced nephrotoxicity through activation of expression levels of SOD2, eNOS, and MMP3.

## Introduction

Nowadays, treatment of hospital infections that occur due to life-threatening multidrug-resistant bacteria, such as *Pseudomonas aeruginosa*, *Acinetobacter baumannii*, and *Klebsiella pneumoniae*, continues to be a serious problem worldwide. Colistin is one of the primary drugs used for the treatment of multidrug-resistant bacteria. However, colistin treatment is highly disadvantageous in particular due to its nephrotoxic effect [1–7]. It is widely accepted that the dosage of colistin is one of the most important variables for determining colistin-induced nephrotoxicity [8–12]. Moreover, pharmacokinetic/pharmacodynamic studies have shown that the effectiveness of colistin treatment at the current recommended dose might not be sufficient for critical patients [13–15]. Critically ill patients have limited treatment options because using high doses of colistin causes nephrotoxicity [16,17]. Finding alternative options is therefore an urgent unmet need in preventing colistin-related nephrotoxicity. Although the exact mechanism behind the induction of nephrotoxicity by colistin is not known, it is suggested that the increased number of reactive oxygen species (ROS) plays an important role in inducing renal tubular apoptosis [18–26]. It has been reported that colistin increased ROS and reduced superoxide dismutase 2 (SOD2), endothelial nitric oxide synthase (eNOS), and glutathione levels neutralize ROS [19,21–26]. Several animal studies suggest that colistin-induced renal damage is reversed, ROS levels are decreased, and apoptosis is reduced when colistin is combined with antioxidant substances including ascorbic acid, vitamin E, melatonin, astaxanthin, and grape seed proanthocyanidin extract [18,19,21,27]. N-acetylcysteine (NAC) is one of the most important antioxidant compounds frequently used to decrease the toxicity of nephrotoxic drugs. The NAC decreases the oxidative stress by binding to ROS. Moreover, it acts as a glutathione precursor, which plays an important role in increasing the glutathione S-transferase activity [28]. Furthermore, several studies have suggested that NAC diminishes the effects of nephrotoxicity caused by elevated ROS as a result of several agents such as vancomycin, gentamicin, ifosfamide, lithium, cisplatin, and contrast agent [29–34]. There are only two studies in the existing literature investigating the effect of NAC on colistin-induced toxicity [21,25]. One of these studies was performed in rats and co-treatment of NAC seemed to ameliorate colistin-induced oxidative stress in kidney cells in rats [21]. An *in vitro* study on neuroblastome-2a cells has shown that colistin-related increase in neuronal apoptosis and ROS was improved by NAC use [25].

Matrix metalloproteinase 3 (MMP3) is an extracellular enzyme capable of extracellular matrix degradation and inhibiting inflammation, and thus it plays an important role in tissue repair [35]. So far, there has been no study that examines the effect of MMP3 in colistin-induced nephrotoxicity.

The exact mechanism of colistin-induced nephrotoxicity and the possible nephroprotective effect of NAC still remain unclear. We have therefore designed this study to investigate the molecular mechanisms of both colistimethate sodium-induced pathological and NAC-induced protective and repair effects.

## Materials and methods

### Animals

Experiments were performed in accordance with National Institutes of Health (NIH) guidelines for the care and use of laboratory animals and approved by local government authorities (Bezmialem Vakif University, Animal Research Ethics Committee (15.02.2013/No: 22)).

A total of 28 male Wistar rats (mean body weight ± the standard deviation, 280 ± 25 gr, 8 weeks old) were obtained from Bezmialem Foundation University Experimental Animals Laboratory in Istanbul, Turkey. Prior to the experiment, the rats were housed individually in metabolic cages for 2 days at a constant temperature (21 ± 1 °C) and humidity (75 ± 5%) controlled facility with a 12-h light/dark cycle and had free access to food and water. In order to measure basal serum creatinine, 1 mL blood was drawn from each rat via the jugular vein under the control of anesthesia with 35 mg/kg ketamine and 5 mg/kg xylazine. In a preliminary study, colistimethate sodium treatment regimens (300 000 IU/kg/day) were evaluated for two different time courses to evaluate the ability of each treatment regime to cause colistin-induced nephrotoxicity in test animals (*n* = 4). 300 000 IU/kg/day of colistimethate sodium was administered intraperitoneally to two of these animals for 7 days, with the daily dosage split to two doses per day; this regimen did not lead to reproducible renal damage based upon histological evaluation. The second regimen with 300 000 IU/kg/day of colistimethate sodium for 10 days on two test animals led to severe kidney lesions; many of these lesions were considered irreversible. Ten-day treatment of colistimethate sodium with 300 000 IU/kg/day dose was used to examine the possible protective effect of NAC.

### Drugs

Colistimethate sodium was provided by Koçak Farma Pharmaceutical Company (Istanbul, Turkey). N-acetylcysteine was provided by Bilim İlaç Pharmaceutical Company (Istanbul, Turkey).

#### Investigation of the interaction between colistimethate sodium and NAC

DrugBank data analysis system was used for this purpose [36]. Potential interaction between NAC and colistimethate sodium was investigated by examining enzymes, receptors, and transporters affected by drugs and the pharmacodynamic and pharmacokinetic properties of drugs in this database system.

### Experiment protocol

The project was carried out with a total of 28 animals. Rats were divided into four groups (*n* = 7 each) and dosed intraperitoneally twice daily for 10 days with (1) 2 ml/kg/day sterile saline (sterile saline group); (2) 300 mg/kg/day NAC (NAC group); (3) 300 000 IU/kg/day colistimethate sodium (colistimethate sodium group); (4) 300 000 IU/kg/day colistimethate sodium + 300 mg/kg/day NAC (colistimethate sodium + NAC group). Drugs were administered to each rat intraperitoneally twice a day with 8 h apart between doses. Drug concentrations were kept identical to the volume on a daily basis for each experimental group. In the colistimethate sodium + NAC group, NAC was administered 30 min prior to injection of the colistin. Urine was collected into a chilling chamber at 4 °C at 24-h intervals for 3 days prior to commencing the treatments (baseline) and on days 1, 5, and 10. Blood was collected via the jugular vein 3 days before initiating the treatments and at the time the rats were sacrificed on day 10. Rats were sacrificed 16 h after the last administered dose of drugs on day 10. The right and left kidneys of each anesthetized rat were removed (90 mg/kg of ketamine and 10 mg/kg of xylazine). The right kidneys were fixed in 10% neutral buffered formalin for histological examination and the left kidneys were frozen in dry ice and stored at −80 °C for apoptosis assay and total oxidant status/total antioxidant status (TAS/TOS) measurement as well as Western blot analysis of eNOS, SOD2, and MMP3 expression levels.

### Biochemical measurements

Blood samples taken from the animals before treatment and during exsanguination were centrifuged at 1687 g for 10 min and stored at −80 °C. Serum creatinine levels were measured through enzyme-linked immunosorbent assay (ELISA) method (Catalog no: MBS883852, MyBioSource, San Diego, CA/Chromate Manager 4300, Palm City, FL). After the urine volume was measured, aliquots were stored at −80 °C to measure the excretion of N-acetyl-β-d-glucosaminidase (NAG) level. The NAG levels in urine were measured as described in the literature [37].

### Histological examination

The formalin-fixed right kidneys were embedded in paraffin; sections (5 μm) were mounted on glass slides and counterstained with hematoxylin-eosin, and periodic acid–Schiff. The samples were examined and histological changes were evaluated with a predefined semiquantitative scoring system by a pathologist who was blinded to the treatment [20]. Lesions formed in the kidneys were pathologically assessed in three grades: mild tubular damage that consists of tubular dilatation, evident nucleus, and a number of pale tubular casts (Grade 1); severe tubular damage that consists of many tubular casts and necrosis of tubular epithelium (Grade 2); and acute cortical necrosis with or without papillary necrosis (Grade 3). Renal histological changes were scored per grades as follows: Grade 1 lesion 1 score; Grade 2 lesion 4 score; and Grade 3 lesion 10 score. The following scores were given according to the percentage of the kidney’s affected area: <1% = 0 score; 1–4% = 1 score; 5–9% = 2 score; 10–19% = 3 score; 20–29% = 4 score; 30–39% = 5 score; and ≥40% = 6 score. Total histological damage score was found by adding the score per grade and the score per percentage of the affected area. Semiquantitative scores (SQS) for renal histological changes were calculated as follows: SQS + 1: mild damage (total score 1–14); SQS + 2: mild-moderate damage (total score 15–29); SQS + 3: moderate damage (total score 30–44); SQS + 4: moderate–severe damage (total score 45–59); and SQS + 5: severe damage (total score 60).

### Apoptosis assay

Detection of DNA fragmentation of apoptotic cells was carried out by terminal transferase biotinylated-dUTP nick end labeling (TUNEL) assay using TUNEL kit (Roche, Basle, Switzerland). Apoptotic cells were counted under a fluorescent microscope on 400 magnifications using Analysis 5 Research Program (Olympus Soft Imaging Solutions, Münster, Germany). In order to obtain the relative average number of apoptotic cells for each animal, apoptotic cells were calculated in five different areas from the cortex and five different areas from the medulla.

## Western blot analysis

The left kidney of each animal was used for Western blot analysis. Tissue samples of each experimental group were pooled and homogenized with radioimmunoprecipitation assay (RIPA) lysis buffer (Thermo Fisher Scientific, Cat No: 89 900, Waltham, MA, USA) containing a protease inhibitor cocktail. Total protein concentrations were measured by using Implen NanoPhotometer P-Class (Implen, Munich, Germany). Equal amounts of protein (40 µg) were fractionated by size in 4–12% NuPAGE electrophoresis, and they were transferred to polyvinylidene fluoride membranes (PVDF) by using iBlot^®^ Dry Blotting System (Invitrogen, Life Technologies Corporation, Waltham, MA, USA). Membranes were blocked in 5% nonfat milk in 50 mM Tris-buffered saline containing 0.1% Tween-20 (TBS-T) at room temperature for 1 h and washed in TBS-T. Then, the blocked membranes were incubated overnight at 4 °C with anti-MMP3 (rabbit monoclonal, 14335S, Cell Signaling Technology, Boston, MA, USA), anti-SOD2 (rabbit monoclonal, 13141S, Cell Signaling Technology, Boston, MA, USA), and anti-eNOS (rabbit polyclonal, NB300–500, Novus Biologicals, Littleton, CO, USA) antibodies. All primary antibodies were used in 1:2500 dilution. The following day, membranes were washed with TBS-T and incubated with horse radish peroxidase conjugated anti-rabbit secondary antibody with 1:2500 dilution (7074S, Cell Signaling Technology, Boston, MA, USA) for 1 h at room temperature. The blots were then developed by using ECL Advanced Western Blotting Detection Kit (GE Healthcare UK Limited, Amersham, UK) and visualized with Bio-Rad ChemiDoc XRS (Bio-Rad Laboratories, Inc, Hercules, CA, USA). Protein amounts were analyzed densitometrically using ImageJ software and the values were corrected and normalized with β-actin blots.

## Measurement of total oxidant and antioxidant status and determination of oxidative stress index (OSI)

The TOS and TAS of the tissue homogenates were determined by using automated analyzer (Chromate Manager 4300, Palm City, FL) as described earlier [38,39]. To determine TOS, intensity of the generated colored complex by ferric ions was measured. Ferric ions were oxidized from ferrous iono-dianisidine complex due to the presence of oxidants, and xylenol orange in an acidic medium was used to evaluate the total oxidant molecules in the samples. The calibration was achieved with hydrogen peroxide. The TOS values were expressed as micromolar hydrogen peroxide equivalents per liter (μmol H_2_O_2_ equiv/L) (Rel Assay Diagnostics, Gaziantep, Turkey).

For TAS measurements, absorbance of colored dianisidyl radicals was determined based upon the rates of the Fenton reactions which trigger free-radical reactions with the production of a hydroxyl radical. The antioxidative effect of the tissue sample against potent free-radical reactions was expressed in terms of mmol equiv/L Trolox (Rel Assay Diagnostics, Gaziantep, Turkey).

With the help of the TOS and TAS values, OSI was calculated with the equation: OSI = ((TOS)/(TAS ×1000)) ×100.

### Statistical evaluation

SPSS 17 (SPSS Inc., Chicago, IL) program was used for statistical analysis. Categorical variables, normally distributed continuous variables, and non-normally distributed continuous variables were referred to as number of cases and percentage, mean ± standard deviation, and median (minimum–maximum) values, respectively. One-way ANOVA [Tukey’s honestly significant difference (HSD) test and Tamhane’s test in comparison of two groups] and Kruskal–Wallis (Mann–Whitney *U*-test with Bonferroni correction in comparison of two groups) tests were used for the comparison of normally distributed variables and non-normally distributed variables among more than two groups, respectively. Repeated measures ANOVA and paired-samples *t*-test were used for the comparison of repetitive measurements in a group. *p* < .05 value was considered as statistically significant.

## Results

A statistically significant increase in the level of post-treatment creatinine was observed in the colistimethate sodium group in comparison with the pretreatment creatinine level (*p* = .001). No significant difference in the level of creatinine between pretreatment and post-treatment among the remaining groups was observed (*p* > .05) (Table 1).

**Table 1.** Comparison of pretreatment and post-treatment serum creatinine levels.

	Pretreatment creatinine (mg/dl)	Post-treatment creatinine (mg/dl)	*p*
Sterile saline group	0.76 ± 0.22	0.86 ± 0.005	.371
N-acetylcysteine group	0.88 ± 0.17	1.01 ± 0.18	.117
Colistimethate sodium group	0.72 ± 0.08	1.25 ± 0.28	.001
Colistimethate sodium + N-acetylcysteine group	0.74 ± 0.13	0.82 ± 0.15	.273

**Figure 1.**
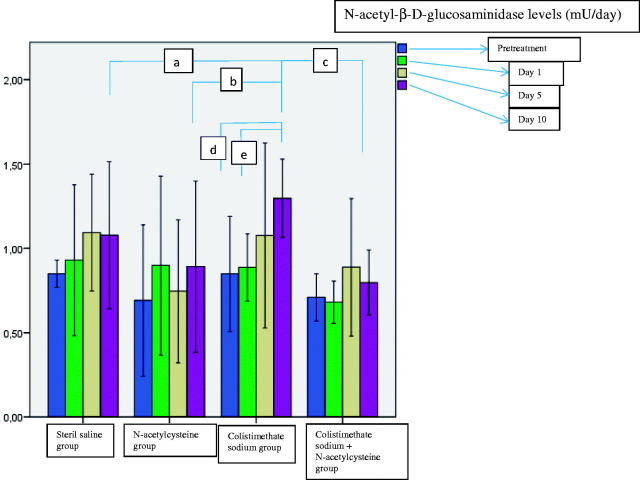
Comparison of urine N-acetyl-β-d-glucosaminidase levels on day 1, 5, and 10 of treatment (^a^*p* = .001 when colistimethate sodium group and sterile saline group were compared on day 10; ^b^*p* = .0001 when colistimethate sodium group and N-acetylcysteine group were compared on day 10; ^c^*p* = .001 when colistimethate sodium group and colistimethate sodium + N-acetylcysteine group were compared on day 10; ^d^*p* = .001 when the 10th day of the treatment was compared with the baseline in the colistimethate sodium group; ^e^*p* = .0001 when the 10th day of the treatment was compared with the first day in the colistimethate sodium group).

The urine NAG levels before treatment and on day 1, 5, and 10 of treatment were measured for each group. There was a significant increase in urine NAG level in the colistimethate sodium-treated rats on day 10 of treatment as compared to the basal value and the first day of treatment (*p* = .001 and .0001 respectively) (Figure 1). Urine NAG levels were higher in the colistimethate sodium group on the 10th day of treatment than in the other groups (Figure 1).

**Figure 2.**
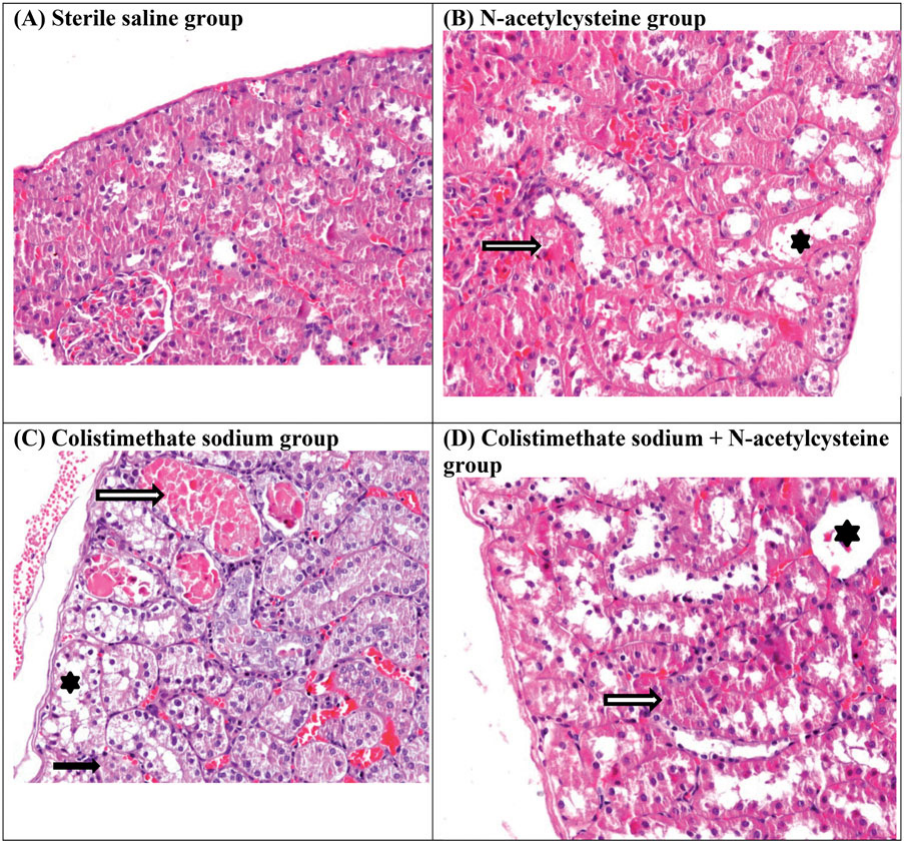
Histological findings of the study: normal kidney structure of the sterile saline group (A), Grade 1 renal histological lesion in N-acetylcysteine group (B), Grade 2 renal histological lesion in colistimethate sodium group (C), Grade 1 renal histological lesion in colistimethate sodium + N-acetylcysteine group (D). Tubular dilatation (asterisk), tubular casts (hollow arrow), and tubular cell necrosis (black arrow) (original magnification ×200).

Colistimethate sodium treatment increased the apoptosis index significantly (Table 2). The kidneys from the colistimethate sodium-treated rats showed a marked renal histological damage score (RHDS) in comparison with other experimental groups (*p* < .05) (Tables 2 and 3, Figure 2). Renal histologic damage score was higher in the NAC group than in the sterile saline group (Table 2).

**Table 2.** TUNEL results and renal histological damage scoring in all experimental groups.

	Sterile saline group	N-acetylcysteine group	Colistimethate sodium group	Colistimethate sodium + N-acetylcysteine group
Rate of apoptosis^a^	45.31 ± 30.49	29.90 ± 27.89	122.62 ± 52.14^b^^,c,d^	66.58 ± 33.32
Semiquantitative score for renal histological changes	0 (0–1)	2 (0–6)	7 (3–10)^e^^,f,g^	2 (2–7)^h^

a Number of apoptotic cells under each – 400 magnifications of light microscopy.

b *p* = .006 as compared with the sterile saline group.

c *p* = .001 as compared with the N-acetylcysteine group.

d *p* = .046 as compared with the colistimethate sodium–N-acetylcysteine group.

e *p* = .001 as compared with the sterile saline group.

f *p* = .001 as compared with the N-acetylcysteine group.

g *p* = .007 as compared with the colistimethate sodium–N-acetylcysteine group.

h *p* = .001 as compared with the sterile saline group.

**Table 3.** The values of renal histological changes among experimental groups.

	The score related to the grade of renal histology^a^	The score related to the size of the affected area in kidney^b^	Semiquantitative score for renal histological changes^c^
Sterile saline group	1	0	1
	0	0	0
	0	0	0
	0	0	0
	0	0	0
	0	0	0
N-acetylcysteine group	1	1	2
	1	1	2
	1	1	2
	1	1	2
	1	1	2
	0	0	0
Colistimethate sodium group	4	3	7
	4	6	10
	4	3	7
	4	3	7
	1	2	3
	1	2	3
	1	2	3
Colistimethate sodium + N-acetylcysteine group	1	1	2
	1	1	2
	1	1	2
	1	1	2
	1	1	2
	1	1	2
	4	3	7

a Grade 1: mild acute tubular damage (tubular dilatation, evident nucleus, very few and pale tubular casts); Grade 2: severe acute tubular damage (tubular cell necrosis and many tubular casts); Grade 3: acute cortical necrosis with or without papillary necrosis. 1 score for Grade 1 change, 4 score for Grade 2 change, 10 score for Grade 3 change.

b The following scores were given according to the percentage of kidney’s affected area: <1%=0 score; 1–4%=1 score; 5–9%=2 score; 10–19%=3 score; 20–29%=4 score; 30–39%=5 score; and ≥40%=6 score.

c Score found by adding the score related to the size of the affected area in kidney and the score related to the degree of renal histology.

**Figure 3.**
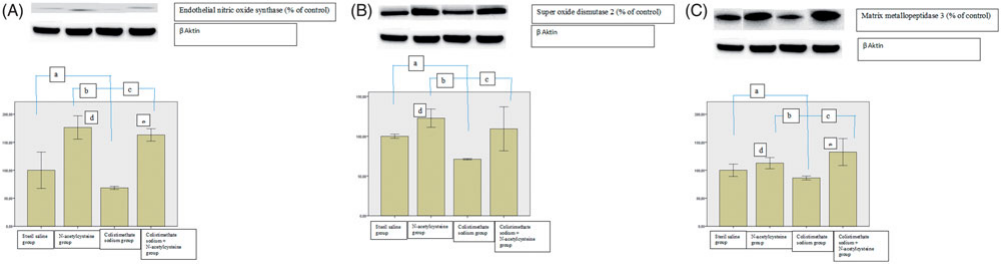
The results of Western blotting analysis in the renal tissue in the sterile saline group, N-acetylcysteine group, colistimethate sodium group, and colistimethate sodium + N-acetylcysteine group: (A) the expression levels of endothelial nitric oxide synthase (a: *p* = .03, compared with the sterile saline group; b: *p* = .0001, compared with the N-acetylcysteine group; c: *p* = .0001, compared with the colistimethate sodium + N-acetylcysteine group; d: *p* = .0001, when N-acetylcysteine group and the sterile saline group were compared; e: *p* = .001, when colistimethate sodium group + N-acetylcysteine group and the sterile saline group were compared); (B) the expression levels of superoxide dismutase 2 (a: *p* = .0001, compared with the sterile saline group; b: *p* = .0001, compared with the N-acetylcysteine group; c: *p* = .002, compared with the colistimethate sodium + N-acetylcysteine group; d: *p* = .001, when N-acetylcysteine group and the sterile saline group were compared); (C) the expression levels of matrix metalloproteinase 3 (a: *p* = .007, compared with the sterile saline group; b: *p* = .0001, compared with the N-acetylcysteine group; c: *p* = .0001, compared with the colistimethate sodium + N-acetylcysteine group; d: *p* = .011, when N-acetylcysteine group and the sterile saline group were compared; e: *p* = .001, when colistimethate sodium group + N-acetylcysteine group and the sterile saline group were compared).

We observed no difference between groups regarding TAS and TOS as well as OSI in the kidneys (*p*-values: .24, .336, and .270, respectively) (Table 4). eNOS, SOD2, and MMP3 levels were lower in the colistimethate sodium group than in all other groups (Figure 3). The eNOS and MMP3 levels were higher in the NAC and colistimethate sodium + NAC groups than in the sterile saline group and colistimethate sodium group (Figure 3). SOD2 levels were higher in the NAC group and colistimethate sodium + NAC group than in the colistimethate sodium group but higher only in the NAC group than in the sterile saline group (Figure 3). By using DrugBank data analysis system, it was seen that there is no interaction between colistimethate sodium and NAC.

**Table 4.** Total oxidant and antioxidant status and determination of oxidative stress index levels in renal tissue of the sterile saline, N-acetylcysteine, colistimethate sodium, and N-acetylcysteine + colistimethate sodium-treated groups.

	Sterile saline group	N-acetylcysteine group	Colistimethate sodium group	Colistimethate sodium + N-acetylcysteine group	*p*
Total oxidant status	3.14 (2.99–3.28)	2.95 (2.91–2.95)	3.10 (2.85–3.43)	3.09 (2.97–3.2)	.240
Total antioxidant status	7.20 (4.65–15.09)	6.88 (2.57–7.58)	4.26 (0.78–6.95)	4.57 (0.05–7.92)	.336
Oxidative stress index	0.24 (0.15–0.46)	0.24 (0.09–0.26)	0.14 (0.003–0.20)	0.15 (0.10–0.26)	.27

## Discussion

In order to show the role of antioxidant substances in preventing colistin-related nephrotoxicity in animal trials, different approaches have been used in the literature regarding colistin dose, duration of treatment, and route of administration. In three studies, colistin was used at a dose of 300 000 IU/kg/day intraperitoneally or intramuscularly during a 6- to 7-day treatment period [19–21]. In other three studies, colistin was also used for 7 days at different doses intravenously [22,26,27]. Additionally, there is controversy in the literature regarding the time required to produce nephrotoxicity with colistin. In one study, it was observed that exposure to colistin at a dose of 300 000 IU/kg/day intramuscularly for 15 days did not cause any histological change in the rat kidney tissue [40]. We prefer to give colistin at a dose of 300 000 IU/kg/day intraperitoneally. But, our study in animal models demonstrated that 300 000 IU/kg/day colistimethate sodium during a 7-day treatment period was not sufficient to induce colistin-related nephrotoxicity, whereas a 10-day treatment period with the same dose of colistimethate sodium was able to induce colistin-related nephrotoxicity in rats.

It is known that the dose of colistin given to rats intraperitoneally, which is 150 000 IU/kg/day, is the maximum therapeutic dose used for humans [8]. In previous studies that examine whether nephrotoxic effects of drugs such as vancomycin and gentamicin are prevented by NAC, it was seen that NAC was used at a dose of 10–200 mg/kg/day intraperitoneally and these doses could prevent histological renal damage [30–34,41]. In two studies performed previously, high doses of NAC such as 500 mg/kg/day were used [42,43]. In only one study that examined the effect of NAC on colistin-related nephrotoxicity, NAC was used at a dose of 150 mg/kg/day intraperitoneally [21]. Patients admitted to hospitals with paracetamol poisoning were treated with the recommended dose of 300 mg/kg/day of NAC [44]. Therefore, 300 mg/kg/day dose regimen of NAC was preferred in order to increase the effectiveness of NAC for the purposes of our study. In our study, NAC was administered intraperitoneally since a high level of elimination occurs during the first passage through the liver when administered orally.

The significant increase in serum creatinine and urine NAG level in the colistimethate sodium-treated group on day 10 showed renal tubular damage when compared to pretreatment values. We did not detect any changes in serum creatinine and urine NAG levels in the colistimethate sodium + NAC group as compared to baseline values, suggesting colistimethate sodium-induced renal tubular damage could be prevented with the use of NAC. Histological examinations showed renal tubular damage in the colistimethate sodium-treated group, whereas renal tubular damage was significantly reduced in the NAC co-treated group. Recently, it has been reported that co-treatment of NAC seemed to ameliorate colistin-induced oxidative stress in kidney cells in rats; however, the cumulative dose of the colistin in this study was very low, and no differences in histological abnormalities were observed between groups, including the colistimethate sodium-treated group [21]. In our study, we observed increased creatinine levels in the colistimethate sodium-treated group. Our study is the first to demonstrate the protective effect of NAC on colistimethate sodium-induced renal histological and biochemical changes. In another study with neuroblastoma-2a cells, colistin administration increased ROS, apoptosis, and caspase-3/7 levels and all these increased levels were diminished with NAC use [25].

**Figure 4.**
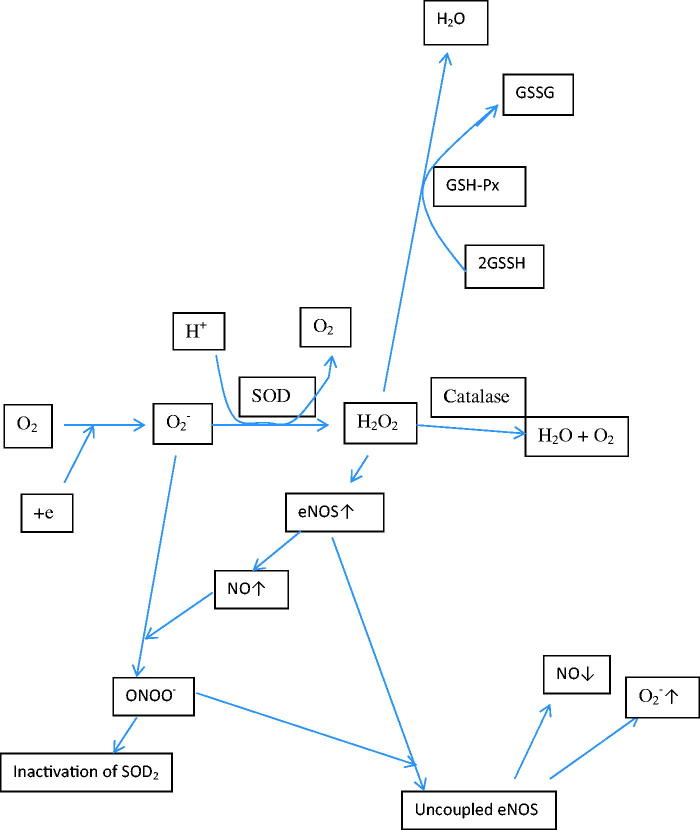
The roles of superoxide dismutase, nitric oxide synthetase, and glutathione in the neutralization of reactive oxygen species formed in the body (O_2_^−^: superoxide; SOD: superoxide dismutase; GSH-Px: glutathione peroxidases; NO: nitric oxide; ONOO^−^: peroxynitrite; H_2_O_2_: hydrogen peroxide; eNOS: endothelial nitric oxide synthase; GSSG: oxidized glutathione; GSSH: reduced glutathione).

It has been suggested that colistin increases ROS and reduces the levels of antioxidant enzymes such as SOD, glutathione, and catalase, and thus it causes neurotoxic and nephrotoxic effects [19,22–26]. Mitochondrial toxicity due to increased ROS has been shown to increase apoptosis in renal cells and neurons, reduce cell viability in tissue cultures, and cause visible cell damage [22–26]. It has been shown *in vitro* and in mice models that increased colistin-induced cell death and reduced level of SOD and glutathione could be reversed by antioxidant substances such as ascorbic acid, curcumin, and lycopene [23,24,26]. In our study, we observed elevated renal cell apoptosis and morphological changes in colistimethate sodium-treated rats, whereas NAC co-treatment reduced apoptosis significantly. In this study, total antioxidant and oxidant capacity was measured in the renal tissue and no difference was found between the groups. The ROS forming as a result of pathological and physiological condition is removed enzymatically and nonenzymatically from the body [45]. The individual measurements of oxidant and antioxidant are preferred to determine the oxidant/antioxidant balance because they provide clearer information [46]. But these methods are time-consuming, labor-intensive, and expensive [47]. Therefore, the methods measuring total oxidant and antioxidant levels have been mostly used in scientific studies due to their simplicity [39,48]. But these methods have some disadvantages: first, each antioxidant molecule makes a different contribution to the total antioxidant status in each different measurement method [47]. We think that the most effective antioxidants for colistin nephrotoxicity might not be measured enough by the selected method in our study. Maybe we should have used at least two different methods for TAS measurements. Second, the levels of some antioxidant molecules like albumin and uric acid can change in concomitant conditions such as renal insufficiency and acute phase response [47]. Renal failure might affect the TAS measurement results in our study. Third, the methods used for TAS measurements can measure only the effect of nonenzymatic antioxidant molecules but not the effect of antioxidant enzymes and metal chelators [47]. Although there was no difference between groups in terms of TAS measurements in our study, antioxidant enzymes levels were lower in the colistimethate sodium group. Fourth, the methods for TOS measurements have low sensitivity and specificity due to the short half-life of ROS [46]. Therefore, it is more plausible to measure the ROS end products level rather than measure the TOS. The disadvantages of the methods for TAS and TOS measurements mentioned above should be the reason why TAS, TOS, and OSI levels are not different between groups in our study. We therefore used Western blot analysis in order to detect specific antioxidant enzymes including expression levels of SOD2 and eNOS in the renal tissue. We observed that the expression levels of SOD2 and eNOS were decreased in the colistimethate sodium-treated group and these effects were reversed and the expression levels of SOD2 and eNOS level were increased in NAC co-treated rats significantly. Our study is the first to show that SOD2 levels decreasing with colistin treatment were reversed by NAC use. The eNOS is an important enzyme for the production of nitric oxide, which has positive effects on the prevention of atherosclerosis [49]. Nitric oxide has been shown to cause vasodilatation, decrease in platelet aggregation and adhesion, decrease in monocyte chemotaxis, decrease in leucocyte adhesion molecules, and inhibition of endothelial apoptosis by inhibiting of ROS [49]. Reducing eNOS levels following colistimethate sodium treatment may have consequences such as increased vasoconstriction and inflammation that may impair renal function. Only two studies have been found in the existing literature regarding the reduction of eNOS levels following colistin administration and the improvement of this condition with NAC and grape seed proanthocyanidin extract treatment [19,21]. The ROS resulting from infection, trauma, and exposure to toxic substances are converted to hydrogen peroxide by SOD_2_ [50] (Figure 4). Hydrogen peroxide increases eNOS synthesis and, consequently, nitric oxide. If SOD2 levels are low, increased superoxide combines with nitric oxide produced by eNOS to form peroxynitrite. Peroxynitrite disrupts eNOS function and inhibits SOD2 enzyme by nitrating tyrosine residues [50]. In light of this information, we think that decreasing levels of SOD2 enzyme might reduce nitric oxide levels by reducing the level of eNOS and disrupting the function. Hydrogen peroxide which is formed by activation of SOD2 enzyme is transformed into water by the effect of glutathione [50]. It is known that NAC has a direct antioxidant effect on ROS and is the source of cysteine in glutathione synthesis [51]. In our study, it appears that NAC might be able to exert an antioxidant effect by increasing SOD2 and eNOS. Tissue damage occurs as a common result of trauma, infections, and autoimmune diseases. As a result of tissue damage, inflammatory cells migrate to the site of the event, followed by tissue repair. The MMP3 is an enzyme that breaks down extracellular matrix elements such as type 4 collagen and laminin and plays an important role in tissue repair processes by regulating inflammation to some extent [35]. The MMP3 has been shown to inhibit monocyte chemotactic protein activity and suppress inflammation by activating mononuclear inflammation-suppressing transforming growth factor-β1 (TGF-β1) [35]. Moreover, MMP3 can both activate and also degrade IL-1β, which plays a role in inflammation [35]. It is shown that fibroblast and keratinocytes on the surface of chronic ulcers play a role in tissue repair by secretion of MMP3 [52]. In our study, we showed that apoptosis and renal tubular cell damage occurred in the kidneys of colistimethate sodium-treated rats. The suppression of inflammation, the destruction of extracellularly matrix, and the beginning of tissue repair process by the effect of MMP3 enzyme in the kidney damaged due to colistin treatment are very important to restore the kidney. Therefore, the MMP3 level may be expected to increase at the onset of the tissue repair process. In our study, colistimethate sodium treatment reduced MMP3 levels, whereas NAC co-treatment increased MMP3 levels significantly. We suggest that this deterioration in tissue repair may contribute to the nephrotoxicity following colistimethate sodium treatment. This is the first study reporting that colistimethate sodium treatment reduces and NAC co-treatment significantly increases the expression level of MMP3 in the renal tissue. It is shown that MMP3 can activate IL-1β and TNF-α (35). The TNF-α and IL1 increase the SOD_2_ levels [53]. It may be possible that the decreasing levels of MMP3 during colistin treatment can reduce the SOD2 enzyme levels by decreasing the TNF-α and IL-1β levels.

The study has some limitations: (1) more than one method could have been used to measure the antioxidant status, (2) the ROS end products could have been measured to determine the oxidant status, and (3) findings from the animal experiments might not be exactly valid for humans.

Here, we provide evidence that antioxidant and free radical scavenger NAC reversed the colistin-induced nephrotoxicity via activation of expression levels of SOD2 and eNOS as well as MMP3.
